# Human papillomavirus-based cervical precancer screening with visual inspection with acetic acid triage to achieve same-day treatments among women living with human immunodeficiency virus infection: test-of-concept study in Ibadan, Nigeria

**DOI:** 10.11604/pamj.2021.40.48.28628

**Published:** 2021-09-20

**Authors:** Olutosin Alaba Awolude, Sunday Oladimeji Oyerinde, Ayokunle Olumuyiwa Ayeni, Isaac Folorunso Adewole

**Affiliations:** 1 Obstetrics and Gynaecology Department, College of Medicine, University of Ibadan, Ibadan, Nigeria,; 2 Infectious Disease Institute, College of Medicine, University of Ibadan, Ibadan, Nigeria,; 3 University College Hospital, Ibadan, Nigeria

**Keywords:** Triage, overtreatment, VIA, same-day, cervical precancer, HPV test, WLHIV, screening

## Abstract

**Introduction:**

cervical precancer screening with same day treatment facilitates maximization of benefits of secondary prevention of cervical cancer. This is particularly important for women living with human immunodeficiency virus (WLHIV) infection because of their exceptional risk for cervical cancer. The availability of HIV programmes in low- and middle-income countries (LMICs) provide unique opportunity for possible introduction “human papillomavirus (HPV) screening followed by visual inspection after application of acetic acid (VIA) with same day treatment of eligible patients”. This study piloted this concept.

**Methods:**

in this prospective, cohort study, 98 WLHIV had HPV and VIA screening for cervical precancer lesions in a HIV clinic in Nigeria. Participants positive to HPV and/or VIA had biopsies from the visible lesions or quadrant of transformation zone. Participants positive to VIA and/or HPV16 or HPV18/45 had same-day thermal ablation treatment and the number of cases documented. The HPV, VIA and scenario of HPV followed by VIA results were compared with histologically confirmed cervical lesion grade 2 or worse statistically.

**Results:**

same day treatment was achieved in 95.0% of eligible cases. Statistically, sensitivity and specificity of VIA was 25.0% and 50.0% and HPV had 95.5% and 75.0%, respectively. In the HPV screening with VIA triage, sensitivity dropped to 45.5% but specificity improved to 100.0%.

**Conclusion:**

triaging HPV positive test with VIA for same-day treatment in cervical precancer screening among PLWHIV looks feasible. The improved specificity will reduce the overtreatment rate, loss to follow-up associated with repeat clinic visits and improve completion of continuum of care.

## Introduction

Invasive cervical cancer (ICC) occurs about 7.9 times more among women living with HIV (WLHIV) than general population [1]. It is the leading cause of cancer death among the 20 million HIV infected women worldwide [2]. In Nigeria, ICC is seen in 2.7 - 6.8% of women living with HIV [3-5].

To reduce this burden of ICC among WLHIV in Nigeria, cervical precancer screening and treatment was prioritized as routine service among WLHIV by Centre for Disease Control and Prevention (CDC) in Nigeria in 2019. The screening uses visual inspection after application of acetic (VIA), an effective and easy to implement method for low- and middle-income countries (LMIC) [6,7]. The method facilitates single visit “screen-and-treat” service and reduces loss to follow-up in the continuum of care [8,9]. However, VIA is limited by performer-dependent results and disparities in sensitivity and specificity to detect high-grade cervical precancer lesions [10]. This necessitated the recommendation of the more sensitive human papillomavirus (HPV) test as the primary screening method in setting that this is feasible [11,12]. The high-risk papillomavirus (hr-HPV) types are carcinogenic and necessary cause of ICC with at least one type detected in 99.7% of cases [10]. However, screening with HPV has the challenge of poor discrimination ability between transient cervical infections and disease state necessitating the need to develop a triaging test to prevent unnecessary treatment [13] and that can, as well, facilitate same day treatment. One of such proposed tests for LMIC is VIA [14].

To achieve this single clinic-visit for HIV and cervical cancer prevention services will require similarly effective and easy to perform HPV testing method with short turn-around time (TAT). The point of service GeneXpert HPV testing platform provides this possibility. The GeneXpert HPV test is a multiplexed, cartridge-based real-time polymerase chain reaction assay with integrated sample preparation that detects 14 hr-HPV types, grouped into 5 channels HPV 16; HPV 18 and/or 45; HPV 31, 33, 35, 52 and/or 58 (P3); HPV 51 and/or 59 (P4); and HPV 39, 56, 66 and/or 68 (P5) [15]. The TAT for the test is about one hour and the platform is available in many HIV and/or TB programs in Nigeria.

However, the efficacy of this two-stage, same day strategy among HIV population remains unclear. This study aimed to pilot the concept of HPV-based primary cervical precancer screening, same day triage of positive cases with VIA for ablative treatment among HIV positive women.

## Methods

**Study design and setting:** this is a prospective, cohort study that evaluated the feasibility of HPV-based primary screening for precancer lesions of the cervix followed by triage of positive cases to determine those that were eligible for same day treatment. The study was conducted between January and March 2020 in a large antiretroviral treatment (ART) programme situated at Infectious Disease Institute, College of Medicine, University of Ibadan, Nigeria.

**Study population:** the study population were women living with HIV (WLHIV). The inclusion criteria were prior sexual exposure irrespective of age, CD4 cells count, viral load, ART treatment status and participation in the routine cervical precancer screening service in the ART programme. Those with previous treatment for cervical diseases and those diagnosed with suspected cervical cancer at presentation for screening were excluded from participation. A consecutive enrolment of purposively sampled 100 participants was done to pilot this test-of-concept study.

**Figure 1:**
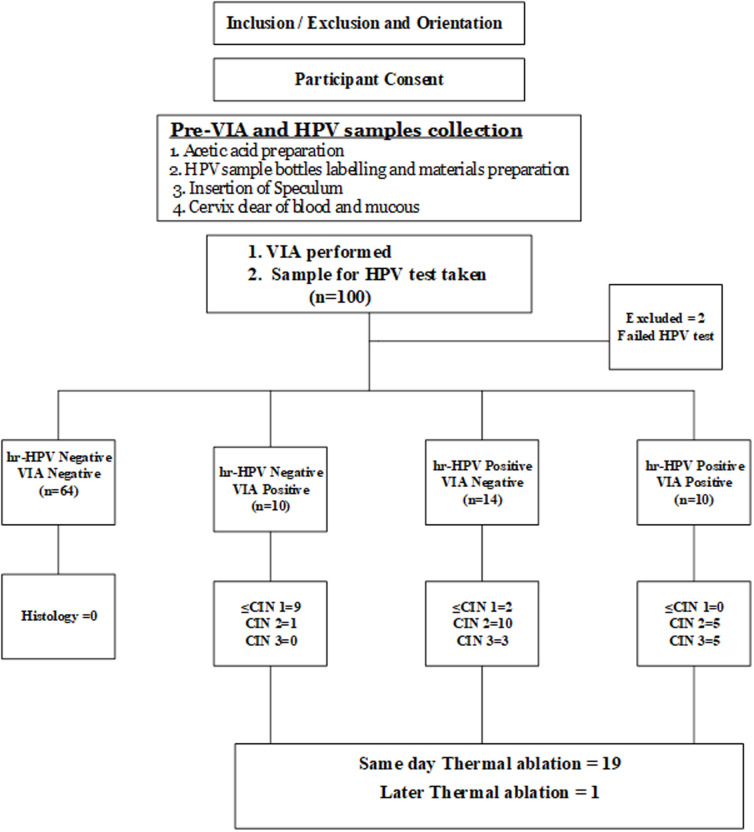
flow chart for screening and treatment algorithms

**Data collection:** each participant underwent routine VIA by trained screening nurse [16] using freshly prepared 3-5% acetic acid applied to the cervix after adequate exposure using appropriate size disposable plastic Cusco’s speculum. The results were read after one minute but not later than 3 minutes and reported according to the IARC criteria [13]. This was followed by collection of cervical cells using a cytobrush (Rovers Medical Devices, The Netherlands), and were placed in PreservCyt medium (Cytyc-Hologic, Marlborough, MA, USA) for the performance of GeneXpert HPV testing in the laboratory within the same facility. In the laboratory, one milliliter of HPV sample was pipetted into the GeneXpert HPV cartridge, which was then slotted into the GeneXpert machine for processing and results obtained approximately one hour. All positive cases to VIA had biopsies of the areas of the cervix with the lesions, while those that were positive for any of the oncogenic strains of HPV, but with absence of a visible lesion on VIA had biopsies from 3, 6, 9 and 12 O’clock of the squamocolumnar junction (Figure 1). Histological confirmation of biopsies was performed at a pathology laboratory by a consultant pathologist with special interest in gynaecology cancers. The consultant was blinded to all clinical and laboratory information including prior HPV and VIA diagnoses. Histological results were classified into five categories: normal (negative for dyplastic lesions), cervicitis, CIN1, CIN2, CIN3 and carcinoma [17]. The end point for evaluating the performance the VIA and HPV test was cervical disease defined as cervical intraepithelial neoplasia 2 and above (CIN 2+) from histological analysis of cervical biopsy specimens. All cases negative to VIA and/or HPV test were scheduled for subsequent routine VIA screening according to the institution protocol. Those participants with positive VIA test and/or those positive for any of the high risk-HPV (hr-HPV) strains had thermal ablation treatment same day after the cervical biopsies. The intention was for women with loop electrosurgical excision procedure (LEEP) eligible lesions or lesions suspicious for cancer on VIA to be managed according to the institutional protocol.

**Definitions:** VIA was considered positive when a well-defined acetowhite area on the external os, the transformation zone or close to the squamo-columnar junction was seen 1 min and not later than 3 minutes after application of 3-5% acetic acid to the cervix. HPV test was considered positive if the gene GeneXpert HPV test detected at least one of the 14 high-risk (hr-HPV) in any of the 5 channels or in combination. Eligibility for same day treatment was considered achieved for any participant positive to HPV 16 and/or HPV 18/45 (alone and in combination with other types) and/or VIA positive. The results of histological examination of CIN 2 and CIN 3 were considered as disease thresholds for this pilot study.

**Statistical analysis:** the statistical analysis for the study was carried out using statistical package for social science statistical (software, version 23.0 (SPSS Inc., Chicago, Illinois). The significance level was set at 5%. Descriptive statistics using frequencies, measures of central tendency (arithmetic mean) and dispersion (standard deviation) were performed for the socio-sexual characteristics of the participants. Sensitivity, specificity, positive predictive value (PPV) and negative predictive value (NPV) were estimated along with their 95% confidence intervals (CIs). This was done for VIA and HPV with histology result as gold standard.

**Ethical consideration:** the study was conducted under the ethical approval of CDC/APIN supported HIV program by APIN ethics committee (IRB number: APIN IRB/19/041-RE.) and University of Ibadan/University College Hospital ethics committee (IRB number: UI/UCH/14/0249). The participants were initially briefed about the study, and informed consent was taken before administering the questionnaire and performing all the tests.

## Results

**General characteristics:** out of the 100 HIV positive women HPV DNA test, 98 had data that were valid for analysis (2 of the participants´ HPV samples analysis failed). The mean age of the participants was 45.8 years [SD±9.2 years and range 23-69 years], 72.4% had at least secondary school education, 71.4% were currently married, 69.2% reported more than one lifetime sexual partner and none reported previous cervical screening services uptake (Table 1).

**Table 1:** general characteristics of the participants

Characteristics	Total (n=98)	HPV positive (n=24)	VIA positive (n=20)
Mean age (years)	45.8 ± 9.2	45.9 ± 8.9	46 ± 10.4
**Marital status**			
Single	6 (6.1%)	2 (8.3%)	1 (5.0%)
Married	70 (71.4%)	15 (62.5%)	14 (70.0%)
Separated/divorced	4 (4.1%)	2 (8.3%)	1 (5.0%)
Widowed	18 (18.4%)	5 (20.8%)	4 (20.0%)
Mean coitarche (years)	20.1 ± 8.7	18.7 ± 2.9	19.4 ± 3.1
Lifetime pregnancies (mean)	4.6 ± 2.5	5.1 ± 3.0	5.4 ± 3.0
Lifetime sexual partners; median	2	3	3

HPV: human papilloma virus; VIA: visual inspection after application of acetic acid

**Cervical pre-cancer screening results:** Table 2 shows accuracy by different primary screening methods, in a 1-visit screen-and-treat scenario. HPV was positive in 24 (24.5%) of the 98 valid results in this test of concept study and VIA positive in 20 (20.4%). The patients positive for HPV had 2 to HPV 16 (these were both in combination with other hr-HPV variants); 2 to HPV 18/45 and 20 to other hr-HPV strains.

**Table 2:** cervical pre-cancer screening results

Primary screening scenario	N=98	Percentage
VIA		
Negative	78	(79.6%)
Positive	20	(20.4%)
HPV		
Negative	74	(75.5%)
Positive	24	(24.5%)
HPV 16 alone	1	
HPV 16 (and VIA positive)	3	
HPV 16 positive	4	(16.7%)
HPV18/45 alone	1	
HPV18/45(and VIA positive)	3	
HPV 18/45 positive	4	(16.7%)
* Others	16	(66.7%)
* Others	Number	HPV panels
P3	8	HPV 31, 33, 35, 52, and/or 58
P3, P4	2	HPV 31, 33, 35, 52, and/or 58; HPV 51 and/or 59
P3, P4, P5	2	HPV 31, 33, 35, 52, and/or 58; HPV 51 and/or 59; HPV 39, 56, 66, and/or 68
P3, P5	1	HPV 31, 33, 35, 52, and/or 58; HPV 39, 56, 66, and/or 68
P4, P5	2	HPV 31, 33, 35, 52, and/or 58; HPV 51 and/or 59
P5	5	HPV 39, 56, 66, and/or 68

HPV: human papilloma virus; VIA: visual inspection after application of acetic acid

**Performances of VIA, HPV and HPV with triage to VIA testing (HPV followed by VIA):** all the 24 participants with positive HPV results and 20 participants that were VIA-positive had biopsies of VIA positive areas and the quadrants of the cervix for those with no obvious VIA positivity to ascertain the validity of HPV screening. Among the 22 participants with CIN 2+, VIA correctly identified the cases in 11/22 (50.0%) and GeneXpert HPV in 21/22 (95.5%). However, with scenario of GeneXpert HPV primary screening with VIA triage for same day treatment identified all the 22 (100.0%) cases with CIN 2+. In identifying cases that were truly negative (specificity), VIA identified 3/12 (25.0%) cases, HPV identified 9/12 (75.0%) cases and in a possible scenario of HPV primary screening and VIA triage identified all the 12 cases (100.0%) that were ≤CIN 1 (Table 3). The overtreatment rate for VIA and HPV in “screen and treat” scenario in this study would have been 47.4% (9/19) 12.5% (3/24), respectively.

**Table 3:** performances of HPV with VIA triage testing

Performance	VIA testing estimate (95%CI)	HPV testing estimate (95%CI)	HPV with VIA triage estimate (95%CI)
**CIN 2+ threshold**			
Sensitivity	50.0% (30.7, 69.3)	95.5% (78.2, 99.2)	45.5% (26.9, 65.3)
Specificity	25.0% (8.9, 53.2)	75.0% (46.8, 91.1)	100.0% (75.8, 100.0)
Positive predictive value	55.0% (34.2, 74.2)	87.5% (69.0, 95.7)	100.0% (72.3, 100.0)
Negative predictive value	21.4% (7.6, 47.6)	90.0% (59.6, 98.2)	50.0% (31.4, 78.5)
Diagnostic accuracy	41.2% (26.4, 57.8)	88.24% (73.4, 95.3)	64.7% (47.9, 78.5)

HPV: human papilloma virus; VIA: visual inspection after application of acetic acid; CI: confidence interval

Feasibility of “same-day” treatment: thirty-three of the 34 participants (97.1%) that were HPV and/or VIA positive had same day cervical biopsies with histology of CIN2+ in 21 cases and the participants that was VIA positive but had biopsy one week later also had CIN2+. Overall, 19 of the 20 (95.0%) eligible participants had same day thermal ablation treatment.

## Discussion

This test of concept of HPV-based cervical precancer screening and triage of positive cases with VIA for same day treatment if eligible is the first attempt in our environment. This study showed that this strategy is feasible in that 95.0% of the eligible participants had thermal ablation treatment same day. This represents the primary intention of this concept study. This is achievable because of the use of HPV test platform with short turn-around time with positive cases triaged with a test that provided immediate results.

The GeneXpert HPV performed quite as well as previously documented in other studies as a screening test [18-20]. Our study showed a moderately high sensitivity (95.5%) and relatively high specificity (75.0%), respectively, for HPV as a standalone test, like what has been shown in other studies conducted in sub-Saharan African countries [21-25]. In addition, its inclusion in the screening strategy resulted in less overtreatment of 47.4% if VIA alone had been used as the screening modality. The sensitivity of GeneXpert HPV of 95.5% was substantially greater than that of VIA of 50.0%. VIA is the commonly used strategy for screen and treat for cervical precancer lesions in most LMIC [26]. In addition, the availability of HPV results from GeneXpert HPV platform within one hour makes it attractive and comparable with VIA in reducing the possibility of loss to follow-up. The aim of an optimal integrated screening and triage strategy should reassure most women that they are at very low risk of cervical cancer, accurately identifying cases that need immediate treatment and reduce the number of those with intermediate risk that requires frequent re-testing for early identification of cases that need treatment. This will be particularly important for the high-risk HIV population. Low-resource settings like ours require this less technical and logistically easy triage system because the capacity for treatment is limited and getting women back for repeat testing is challenging or impossible. The availability of Xpert HPV platform and VIA testing method ensure such opportunity.

Our study found that the gain in specificity by adding VIA to HPV testing was at the expense of loss of sensitivity. This might be a problem in mass screening program. However, in a facility based routine screening, this might not be of particular concern especially with a positive predictive value of the sequential testing of 100%.

A major strength of this work is the use of histology as reference standard and reporting by an experienced pathologist with many years of collaboration [27-29], unlike most reports evaluating accuracy of VIA for cervical cancer. In previous studies conducted worldwide under screening conditions, the gold standard was colposcopy and colposcopy-guided biopsy of abnormal areas, an approach that has proven to yield errors in disease recognition in that there can be overestimation of test accuracy due to high correlation between VIA and colposcopy results [30-32]. The ability to perform thermal ablation for 19/20 (95.0%) of the participants believed to be eligible for treatment same day demonstrated the feasibility of this concept. This high overtreatment rate will be avoided in HPV/VIA sequential testing because all the histologically confirmed 12 cases of CIN2+ were identified by this testing strategy.

HPV allowed adequate detection of CIN2+ in our analyses, resulting in a sensitivity of 95.5%% (95% CI: 79.6 - 100%) and a specificity of 75.0% (95% CI: 70.6 - 78.1%). This sensitivity is comparable with what have been reported in some other studies in Africa among HIV population [17]. In addition, GeneXpert HPV offers simplicity of testing, flexibility with non-batching of individual samples and rapid results to facilitate same day treatment for eligible patients [33]. Although sensitivity of HPV is consistently higher, the specificity, however, is usually low. When the specificity of a test is low, its clinical use can result in increased numbers of follow-up assessments, psychological concerns and unnecessary treatment. Therefore, reducing the number of false positive cases while ensuring adequate identification of true cases is essential for the effectiveness of a screening program which our current test of concept is suggesting. Triaging is a sequential testing to improve the specificity of a screening strategy with a second test performed in women screened positive in the first test [34]. While VIA fails to achieve this desired level of specificity as a standalone test, it achieved it in sequential strategy after initial screening positivity with HPV test as recommended by WHO [35]. Though, the loss of sensitivity in this VIA triage strategy of about 50% to detect CIN 2+ has been documented in other similar studies [14,34,36] and the resultant 25% increase in specificity is a plus in reducing failed diagnosed CIN 2+ among this high-risk population. Besides, accuracy of sequential testing was higher than that of VIA as a standalone screening tool in our study suggesting that VIA as a standalone test for “screen and treat”, as advocated for LMIC, will only be useful to reduce the rate of needless treatment if only its sensitivity and specificity can be improved.

Our study was limited by the small size of the study population. Accuracy estimates based on a larger number of women would have provided more robust and probably generalizable results. In addition, the reference standard of cervical biopsy was not performed in participants with negative HPV or VIA test results, suggesting that limiting the biopsies to women based on HPV and/or VIA-positive results identified most women with CIN2+ in only this subset of participants (34/98; 34.7%).

## Conclusion

This study demonstrated the feasibility of same day ablative treatment of eligible patients from HPV-based primary screening for cervical cancer with VIA triage. However, there is need to conduct a larger study to validate this concept. Such larger studies with well calculated sample size and adequate power will provide insight into some of the other issues that have come up from this pilot and make the results obtained more generalizable. Other potential benefits will include establishment of more cost-effective screening strategy of longer screening intervals for screen negative women.

### What is known about this topic

- Cervical precancer screening using HPV test is the most sensitivity method;
- There is need to combine HPV screening with another screening method for effective identification of those that need treatment to avoid overtreatment with associated disadvantage;
- VIA is one of such methods advocated for LMIC where logistics of cytology remain difficult to overcome.

### What this study adds

- The screening for cervical precancer lesions with HPV and triaging to same-day treatment using VIA in an HIV programme is feasible;
- The accuracy to identify patients that do not require treatment was improved by two stage screening using HPV followed by VIA thereby eliminating the problem of overtreatment;
- Overcoming the problem of loss to follow up with associated poor optimization of benefits of screening for precancer lesions of the cervix looks achievable with this strategy.
